# On solutions for a generalized Navier–Stokes–Fourier system fulfilling the entropy equality

**DOI:** 10.1098/rsta.2021.0351

**Published:** 2022-11-14

**Authors:** Anna Abbatiello, Miroslav Bulíček, Petr Kaplický

**Affiliations:** ^1^ Department of Mathematics ‘G. Castelnuovo’, Sapienza University of Rome, Piazzale Aldo Moro 5, 00185 Rome, Italy; ^2^ Faculty of Mathematics and Physics, Charles University, Sokolovská 83, 18675 Prague 8, Czech Republic

**Keywords:** incompressible fluid, heat conducting fluid, stability, inhomogeneous temperature, non-Newtonian fluid, entropy equality, renormalized solution

## Abstract

We consider a flow of a non-Newtonian heat conducting incompressible fluid in a bounded domain subjected to the homogeneous Dirichlet boundary condition for the velocity field and the Dirichlet boundary condition for the temperature. In three dimensions, for a power-law index greater or equal to 11/5, we show the existence of a solution fulfilling the entropy equality. The entropy equality can be formally deduced from the energy equality by renormalization. However, such a procedure can be justified by the DiPerna–Lions theory only for p>5/2. The main novelty is that we do not renormalize the temperature equation, but rather construct a solution which fulfils the entropy equality.

This article is part of the theme issue ‘Non-smooth variational problems and applications’.

## 1. Formulation of the problem

We study the generalized Navier–Stokes–Fourier system

1.1*a* $$\begin{array}{rl} & \partial_{t}v+\mathrm{div}(v⊗v)-\mathrm{div}\text{S}+\nabla \pi =0, \end{array}$$

1.1*b* $$\begin{array}{rl} & \mathrm{div}v=0, \end{array}$$

1.1*c* $$\begin{array}{rl} \;\mathrm{and}\; & \partial_{t}(c_{\nu }\vartheta )+\mathrm{div}(c_{\nu }\vartheta v)+\mathrm{div}q=\text{S}:\text{D}v, \end{array}$$

in $Q:=(0,T)\times \Omega \subset (0,+\infty )\times R^{3}$ with Ω being an open, connected bounded set with Lipschitz boundary. The system (1.1*a*)–(1.1*c*) is completed by the following boundary conditions:

1.2 $$v=0,\;\vartheta =\vartheta_{b}\;\text{on}\;(0,T)\times \partial \Omega ,$$

where the function $\vartheta_{b}=\vartheta_{b}(x)$ is a non-trivial function of the position, and the initial conditions are given as follows:

1.3 $$v=v_{0},\;\vartheta =\vartheta_{0}\;\text{in}\;\{0\}\times \Omega .$$

Here, $v:Q\to R^{3}$ denotes the velocity field, $Dv:=(\nabla v+{\left(\nabla v\right)}^{t})/2$ is the symmetric part of the velocity gradient ∇v, π:Q→R is the pressure, ϑ:Q→R is the temperature, $S:Q\to R_{\mathrm{sym}}^{3\times 3}$ denotes the viscous part of the Cauchy stress tensor and $q:Q\to R^{3}$ is the heat flux.

Concerning the material parameters, the constant $c_{\nu }>0$ in (1.1*c*) denotes the heat capacity and, for simplicity and without losing the generality, we set $c_{\nu }\equiv 1$ in what follows. The heat flux q is represented by the Fourier law:

1.4 q=−κ(ϑ)∇ϑ,

with the heat conductivity κ:R→(0,+∞) being a continuous function of the temperature satisfying, for all ϑ∈(0,+∞) and for some $0<\underline{\kappa },\bar{\kappa }<+\infty ,$

1.5 $$0<\underline{\kappa }\leq \kappa (\vartheta )\leq \bar{\kappa }<+\infty .$$

We assume that $S=S^{*}(\vartheta ,Dv)$, where $S^{*}:(0,\infty )\times R_{\mathrm{sym}}^{3\times 3}\to R_{\mathrm{sym}}^{3\times 3}$ is a continuous mapping fulfilling for some p≥11/5, some $0<\underline{\nu }$, $\bar{\nu }<+\infty$ and for all $\vartheta \in R_{+}$, $D_{1},D_{2}\in R_{\mathrm{sym}}^{3\times 3}$

1.6*a* $$({\text{S}}^{*}(\vartheta ,{\text{D}}_{1})-{\text{S}}^{*}(\vartheta ,{\text{D}}_{2})):({\text{D}}_{1}-{\text{D}}_{2})\geq 0$$

and

1.6*b* $${\text{S}}^{*}(\vartheta ,{\text{D}}_{1}):{\text{D}}_{1}\geq \underline{\nu }|{\text{D}}_{1}{|}^{p}-\bar{\nu },\;|{\text{S}}^{*}(\vartheta ,{\text{D}}_{1})|\leq \bar{\nu }{\left(1+|{\text{D}}_{1}|\right)}^{p-1},\;{\text{S}}^{*}(\vartheta ,0)=0.$$

Note that the prototypic relation $S∼\nu (\vartheta )|Dv{|}^{p-2}Dv$ falls into the class (1.6). Also to simplify further the notation and assumptions, we introduce a unique $\hat{\vartheta }$, which is a solution to

1.7 $$-\mathrm{div}(\kappa (\hat{\vartheta })\nabla \hat{\vartheta })=0\;\text{in }\Omega ,\;\hat{\vartheta }=\vartheta_{b}\;\text{on }\partial \Omega ,$$

and prescribe all assumptions in terms of $\hat{\vartheta }$.

The key motivation of this article is the qualitative analysis of the system (1.1). More precisely, in a three-dimensional setting, the existence of a weak solution to (1.1) was firstly proved for p≥11/5 in [1], see also the related work [2]. Later, for p∈(9/5,11/5) and slightly different boundary conditions, the existence of a weak solution was established in [3], and see also [4] for a more complicated model, with one proviso. The identity (1.1*c*) was replaced by the inequality, which in terms of the entropy

1.8 η=log⁡ϑ

can be rewritten into the so-called entropy inequality:

1.9 $$\partial_{t}\eta +\mathrm{div}(\eta v)+\mathrm{div}\left(\frac{\text{q}}{\vartheta }\right)\geq \frac{\text{S}:\text{D}\text{v}}{\vartheta }+\kappa (\vartheta )\frac{|\nabla \vartheta {|}^{2}}{\vartheta^{2}}.$$

A natural question, one may ask, is how regular is the solution (ϑ,v). However, looking on equation (1.1*c*), we see that system (1.1) has the so-called critical growth on the right-hand side, and there is no *a priori* regularity theory available. The main reason is that the right-hand side of (1.1*c*) is just integrable. On the other hand, it seems to be extremely important to justify certain renormalization of equation (1.1*c*) since the renormalized solution can typically have better properties on one hand and on the other hand one may use the renormalization to rigorously justify arguments based on the notion of classical solution, e.g. when studying the stability theory, see [5]. Unfortunately, the classical technique of DiPerna and Lions, see ([6], Lemma II.1 and Theorem II.1), can be used only for $p>\frac{5}{2}$ here. Therefore, for p∈[11/5,5/2], we need to change the methods and results used in [1,3,7] significantly. Thus, our main goal is to prove the existence of a weak solution, which satisfies (1.9) with the *equality sign* and also to show that the temperature is continuous with respect to time into the topology of $L^{1}(\Omega )$, which is the natural function space. Then, inspired by [5], we know that one can renormalize (1.9) by a properly chosen set of functions. Indeed, in the standard approach of renormalization, based just on (1.1*c*), one needs (due to the commutator lemma) that $\vartheta \in L^{p^{\prime }}(Q)$. Unfortunately, this is true only for $p>\frac{5}{2}$. Therefore, we introduce (1.9) with equality sign, and then to renormalize it, one just requires $\eta \in L^{p^{\prime }}(Q)$, which is true for any p>1. Our result can be understood as a starting point and the cornerstone of further analysis, which in principle requires very special test functions, e.g. the regularity theory, the stability theory and so on, in various models of incompressible heat conducting fluids. Note that the bound $p\geq \frac{11}{5}$ then does not come from the heat equation but is a consequence of the structure of (1.1*a*) and is closely related to the difference of a notion of solution introduced in [1,7]. More precisely, for our theory, it is necessary to obtain at least weak sequential compactness of the term S:Dv arising on the right-hand side of (1.1*c*), which is known only in the case $p\geq \frac{11}{5}$.

## 2. Rigorous statement of the main result

In what follows, we use the standard notation for the Lebesgue, the Sobolev and the Bochner spaces and endow them with standard norms. The symbol $C_{0}^{\infty }$ is reserved for smooth compactly supported functions, and the function spaces related to the incompressible setting are denoted by $W_{0,\mathrm{div}}^{1,p}:=\{v\in W_{0}^{1,p}(\Omega ;R^{3});\;\mathrm{div}v=0\}$ and $L_{0,\mathrm{div}}^{2}$ denotes the closure of $W_{0,\mathrm{div}}^{1,2}$ in $L^{2}$ topology. Duality pairing between $W_{0,\mathrm{div}}^{1,p}$ and their duals is denoted ⟨⋅,⋅⟩.

Next, we postulate the assumptions on data. Recall, we assume that q and $S^{*}$ satisfy (1.4)–(1.6). For initial and boundary data, we consider

2.1 $$v_{0}\in L_{0,\mathrm{div}}^{2},\;\vartheta_{0}\in L^{1}(\Omega ),\;\hat{\vartheta }\in W^{1,2}(\Omega )\cap L^{\infty }(\Omega ),$$

where $\hat{\vartheta }$ is the solution to (1.7). Hence, we transferred all assumptions on the boundary behaviour of $\vartheta_{b}$ to the uniquely defined $\hat{\vartheta }$. Finally, we suppose that

2.2 $$\mu :=\min\{{\mathrm{ess}\;\inf}_{x\in \Omega }\hat{\vartheta }(x),\;{\mathrm{ess}\;\inf}_{x\in \Omega }\vartheta_{0}(x)\}>0.$$

The main result of this article is presented as follows.

**Theorem 2.1 (Existence of a solution fulfilling entropy equality).**

*Let* $\Omega \subset R^{3}$ *be a bounded domain with Lipschitz boundary. Assume that* $S^{*}$ *and* κ *satisfy* (1.5)–(1.6) *with* p≥11/5. *Then for any data* $v_{0},\vartheta_{0},\hat{\vartheta }$ *fulfilling* (2.1)–(2.2), *there exists a quadruplet* (v,S,ϑ,η) *such that*

2.3 $$\begin{array}{rl} & v\in C([0,T];L_{0,\mathrm{div}}^{2})\cap L^{p}(0,T;W_{0,\mathrm{div}}^{1,p}), \end{array}$$

2.4 $$\begin{array}{rl} & \partial_{t}v\in L^{p^{\prime }}(0,T;{\left(W_{0,\mathrm{div}}^{1,p}\right)}^{*}),\;\text{S}\in L^{p^{\prime }}(Q;R^{3\times 3}), \end{array}$$

2.5 $$\begin{array}{rlr} & \vartheta \in C([0,T];L^{1}(\Omega )),\;{\left(\vartheta \right)}^{\alpha }\in L^{2}(0,T;W^{1,2}(\Omega )) & \text{for any}\;\alpha \in \left(0,\frac{1}{2}\right), \end{array}$$

2.6 $$\begin{array}{rlr} & \vartheta \in L^{r}(Q) & \text{for any}\;r\in \left[1,\frac{5}{3}\right), \end{array}$$

2.7 $$\begin{array}{rlr} & \vartheta -\hat{\vartheta }\in L^{s}(0,T;W_{0}^{1,s}(\Omega )) & \text{for any}\;s\in \left[1,\frac{5}{4}\right) \end{array}$$

2.8 $$\begin{array}{rlr} \;\mathrm{and}\; & \eta \in L^{2}(0,T;W^{1,2}(\Omega ))\cap L^{q}(Q) & \text{for any}\;q\in [1,+\infty ), \end{array}$$

and satisfying (1.1) *and* (1.9) *in the following sense*:

*Momentum equation: The Cauchy stress is of the form* $S=S^{*}(\vartheta ,Dv)$ *a.e. in* Q, *the initial datum fulfils* $v(0)=v_{0}$ *and for all* $w\in L^{p}(0,T;W_{0,\mathrm{div}}^{1,p})$
  2.9 $$\int_{0}^{T}\left\langle \partial_{t}v,w\right\rangle \;dt+\int_{0}^{T}\int_{\Omega }\text{S}:\text{D}w\;\;dx\;dt=\int_{0}^{T}\int_{\Omega }(v⊗v):\text{D}w\;\;dx\;dt.$$
*Internal energy balance: Temperature satisfies the minimum principle* ϑ≥μ a.e. in Q, *the initial condition fulfils* $\vartheta (0)=\vartheta_{0}$ *and for all* $\varphi \in C_{0}^{\infty }((-\infty ,T)\times \Omega )$
  2.10 $$\begin{array}{rl} & \;-\int_{0}^{T}\int_{\Omega }\vartheta \partial_{t}\varphi \;\;dx\;dt-\int_{0}^{T}\int_{\Omega }\vartheta v\cdot \nabla \varphi \;\;dx\;dt+\int_{0}^{T}\int_{\Omega }\kappa (\vartheta )\nabla \vartheta \cdot \nabla \varphi \;\;dx\;dt\\ & \;\;=\int_{0}^{T}\int_{\Omega }\text{S}:\text{D}v\;\varphi \;\;dx\;dt+\int_{\Omega }\vartheta_{0}\varphi (0)\;\;dx. \end{array}$$
*Entropy equation: Entropy is given as* η=ln⁡ϑ a.e. in Q, $\eta_{0}:=\ln\vartheta_{0}$, *and for all* $\varphi \in C_{0}^{\infty }((-\infty ,T)\times \Omega ),$
  2.11 $$\begin{array}{rl} & \;-\int_{0}^{T}\int_{\Omega }\eta \partial_{t}\varphi \;\;dx\;dt-\int_{0}^{T}\int_{\Omega }\eta \;v\cdot \nabla \varphi \;\;dx\;dt+\int_{0}^{T}\int_{\Omega }\kappa (\vartheta )\nabla \eta \cdot \nabla \varphi \;\;dx\;dt\\ & \;\;=\int_{0}^{T}\int_{\Omega }\frac{1}{\vartheta }\;\text{S}:\text{D}v\;\varphi \;\;dx\;dt+\int_{0}^{T}\int_{\Omega }\kappa (\vartheta )\frac{|\nabla \vartheta {|}^{2}}{{\left(\vartheta \right)}^{2}}\;\varphi \;\;dx\;dt+\int_{\Omega }\eta_{0}\;\varphi (0)\;\;dx. \end{array}$$

## 3. Proof of theorem 2.1

The existence proof relies on the methods developed in [3], and a large part of the proof is identical. Therefore, we omit unnecessary details and focus mainly on the new aspects of the proof, i.e. on the proof of entropy equality (2.11).

Hence, following [3] (compare also with [1], where a different approach is used), we introduce a basis ${\left\{w_{j}\right\}}_{j=1}^{\infty }$ of the space $W^{3,2}(\Omega ;R^{3})\cap W_{0,\mathrm{div}}^{1,p}$ that is orthonormal in $L_{0,\mathrm{div}}^{2}$, see ([8], appendix A.4). Next, for given initial conditions $v_{0}$ and $\vartheta_{0}$, we denote $v_{0}^{n}$ the projection of $v_{0}$ onto the subspace $\left[w_{1},\ldots ,w_{n}\right]$, and $\vartheta_{0}^{n}\in L^{2}(\Omega )$, fulfilling $\vartheta_{0}^{n}\geq \mu$ a.e. in Ω, is the regularization of $\vartheta_{0}$ such that

3.1 $$v_{0}^{n}\to v_{0}\;\text{strongly in}\;L_{0,\mathrm{div}}^{2}\text{ as }n\to +\infty$$

and

3.2 $$\vartheta_{0}^{n}\to \vartheta_{0}\;\text{strongly in}\;L^{1}(\Omega )\;\text{as}\;n\to +\infty .$$

Then, for every n∈N, we can find a triple $\left(v^{n},\vartheta^{n},S^{n}\right)$, such that $v^{n}\in W^{1,2}((0,T),W^{3,2}(\Omega ;R^{3})\cap W_{0,\mathrm{div}}^{1,p})$, $\vartheta^{n}\in C([0,T];L^{2}(\Omega ))\cap L^{2}((0,T);W^{1,2}(\Omega ))\cap W^{1,2}((0,T);{\left(W_{0}^{1,2}(\Omega )\right)}^{*})$ and $S^{n}\in L^{\infty }(Q)$ and such that for a.e. t∈(0,T)

3.3 $$\int_{\Omega }[\partial_{t}v^{n}\cdot w_{j}-(v^{n}⊗v^{n}):\nabla w_{j}+{\text{S}}^{n}:\text{D}w_{j}]\;\;dx=0\;\text{for all}\;j=1,\ldots ,n,$$

and for all $\psi \in L^{2}(0,T;W_{0}^{1,2}(\Omega ))$

3.4 $$\begin{array}{rl} & \;\int_{0}^{T}\langle \partial_{t}\vartheta^{n},\psi \rangle \;dt+\int_{0}^{T}\int_{\Omega }[-\vartheta^{n}v^{n}\cdot \nabla \psi +\kappa (\vartheta^{n})\nabla \vartheta^{n}\cdot \nabla \psi ]\;\;dx\;dt\\ & \;\;=\int_{0}^{T}\int_{\Omega }{\text{S}}^{n}:\text{D}v^{n}\psi \;\;dx\;dt. \end{array}$$

In addition, $v^{n}$ and $S^{n}$ are given by

3.5 $$v^{n}=\sum_{i=1}^{n}c_{i}^{n}(t)w_{i}(x)\;\text{ and }\;{\text{S}}^{n}={\text{S}}^{*}(\vartheta^{n},\text{D}v^{n}),$$

the initial conditions $v^{n}(0,\cdot )=v_{0}^{n}$, $\vartheta^{n}(0,\cdot )=\vartheta_{0}^{n}$ are satisfied (note $v^{n}$ and $\vartheta^{n}$ are continuous into the topology of $L^{2}$, and thus, it makes sense to talk about the initial value) and $\vartheta^{n}$ attains the boundary conditions, i.e. $\vartheta_{|\partial \Omega }^{n}=\hat{\vartheta }$ and fulfils the minimum principle, i.e. $\vartheta^{n}\geq \mu$ a.e. in Q.

Then, following [3] and defining ${\tilde{\vartheta }}^{n}:=\vartheta^{n}-\hat{\vartheta }$, it is rather standard to deduce the following n-independent *a priori* estimates valid for all r∈[1,5/3), s∈[1,5/4) and α∈(0,1/2):

3.6 $$\begin{array}{rl} & \;\|v^{n}{\|}_{L^{\infty }(0,T;L_{0,\mathrm{div}}^{2})}+\|v^{n}{\|}_{L^{p}(0,T;W_{0,\mathrm{div}}^{1,p})}+\|{\text{S}}^{n}{\|}_{L^{p^{\prime }}(Q)}+\|v^{n}{\|}_{L^{\frac{5p}{3}}(Q)}\leq C, \end{array}$$

3.7 $$\begin{array}{rl} & \;\|{\tilde{\vartheta }}^{n}{\|}_{L^{\infty }(0,T;L^{1}(\Omega ))}+\|\partial_{t}\vartheta^{n}{\|}_{L^{1}(0,T;{\left(W_{0}^{1,10}(\Omega )\right)}^{*})}+\|\partial_{t}v^{n}{\|}_{L^{p^{\prime }}(0,T;{\left(W_{0,\mathrm{div}}^{1,p}\right)}^{*})}\leq C \end{array}$$

3.8 $$\begin{array}{rl} \;\mathrm{and}\; & \;\|{\left(\vartheta^{n}\right)}^{\alpha }{\|}_{L^{2}(0,T;W^{1,2}(\Omega ))}+\|{\tilde{\vartheta }}^{n}{\|}_{L^{r}(Q)}+\|{\tilde{\vartheta }}^{n}{\|}_{L^{s}(0,T;W_{0}^{1,s}(\Omega ))}\leq C(\alpha ,r,s). \end{array}$$

This is the starting point of the proof. It is rather sketchy, but it does not contain any essentially new information.

### (a) Limit in momentum and energy equations as n→+∞

By virtue of the established uniform estimates (3.6)–(3.8) and employing the Aubin–Lions compactness lemma, we can extract a subsequence that we do not relabel, and we can find (v,ϑ,S) such that

3.9 $$\begin{array}{rl} v^{n}{⇀}^{*}v & \;\text{ weakly-* in }L^{\infty }(0,T;L_{0,\mathrm{div}}^{2}), \end{array}$$

3.10 $$\begin{array}{rl} v^{n}⇀v & \;\text{ weakly in }L^{p}(0,T;W_{0,\mathrm{div}}^{1,p})\cap W^{1,p^{\prime }}(0,T;{\left(W_{0,\mathrm{div}}^{1,p}\right)}^{*}), \end{array}$$

3.11 $$\begin{array}{rl} {\text{S}}^{n}⇀\text{S} & \;\text{ weakly in }L^{p^{\prime }}(Q;R^{3}), \end{array}$$

3.12 $$\begin{array}{rl} v^{n}\to v & \;\text{ strongly in }L^{q}(Q;R^{3})\text{ for any }q\in \left[1,\frac{5p}{3}\right),\text{ and a.e. in }Q, \end{array}$$

3.13 $$\begin{array}{rl} {\left(\vartheta^{n}\right)}^{\alpha }⇀{\left(\vartheta \right)}^{\alpha } & \;\text{ weakly in }L^{2}(0,T;W^{1,2}(\Omega ))\text{ for any }\alpha \in \left(0,\frac{1}{2}\right), \end{array}$$

3.14 $$\begin{array}{rl} \vartheta^{n}\to \vartheta & \;\text{ strongly in }L^{r}(Q)\text{ for any }r\in [1,5/3),\text{ and a.e. in }Q \end{array}$$

3.15 $$\begin{array}{rl} \;\mathrm{and}\;\vartheta^{n}-\hat{\vartheta }⇀\vartheta -\hat{\vartheta } & \;\text{ weakly in }L^{s}(0,T;W_{0}^{1,s}(\Omega ))\text{ for any }s\in \left[1,\frac{5}{4}\right). \end{array}$$

In addition, by using (3.14) and the first part of the uniform estimate (3.7), we have that

3.16 $$\vartheta \in L^{\infty }(0,T;L^{1}(\Omega )).$$

Now we are in the position to analyze the limit of formulations (3.3)–(3.4). Indeed, by virtue of the convergence results (3.10)–(3.12), we may follow [9] to take the limit in formulation (3.3) to deduce that for all $w\in L^{p}(0,T;W_{0,\mathrm{div}}^{1,p})$

3.17 $$\int_{0}^{T}\left\langle \partial_{t}v,w\right\rangle \;dt+\int_{0}^{T}\int_{\Omega }\text{S}:\text{D}w\;\;dx\;dt=\int_{0}^{T}\int_{\Omega }(v⊗v):\text{D}w\;\;dx\;dt.$$

In addition, we have $v\in C([0,T];L_{0,\mathrm{div}}^{2})$ and $v(0,\cdot )=v_{0}$. To complete the proof of (2.9), it remains to show

3.18 $$\text{S}={\text{S}}^{*}(\vartheta ,\text{D}v)\;\text{ a.e. in }Q.$$

Next, thanks to (3.12)–(3.15), one may follow a standard procedure (see, e.g. [7]) and let n→∞ in (3.4) to show that for all $\psi \in C_{0}^{\infty }((-\infty ,T)\times \Omega ),$ there holds (compare with (2.10))

3.19 $$\begin{array}{rl} -\int_{0}^{T} & \;\int_{\Omega }\vartheta \partial_{t}\psi \;\;dx\;dt-\int_{0}^{T}\int_{\Omega }\vartheta v\cdot \nabla \psi \;\;dx\;dt+\int_{0}^{T}\int_{\Omega }\kappa (\vartheta )\nabla \vartheta \cdot \nabla \psi \;\;dx\;dt\\ & \;=\int_{0}^{T}\int_{\Omega }\text{S}:\text{D}v\;\psi \;\;dx\;dt+\int_{\Omega }\vartheta_{0}\psi (0)\;\;dx, \end{array}$$

provided that we show

3.20 $${\text{S}}^{n}:\text{D}v^{n}⇀\text{S}:\text{D}v\;\text{weakly in}\;L^{1}(Q).$$

Obviously, we also have ϑ≥μ a.e. in Q. It remains to show (3.18) and (3.20). Note that both of them are consequences of the Minty method. Indeed, it follows from (3.3) that (using the fact that $\mathrm{div}v^{n}=0$ and (3.10))

$$\begin{array}{rl} & \;\underset{n\to \infty }{lim sup}\int_{0}^{T}\int_{\Omega }{\text{S}}^{n}\cdot \text{D}v^{n}\;\;dx\;dt=\underset{n\to \infty }{lim sup}-\int_{0}^{T}\langle \partial_{t}v^{n},v^{n}\rangle \;dt\\ & \;\;=\underset{n\to \infty }{lim sup}\left(-\int_{0}^{T}\langle \partial_{t}(v^{n}-v),v^{n}-v\rangle \;dt-\int_{0}^{T}\langle \partial_{t}v,v^{n}-v\rangle \;dt-\int_{0}^{T}\langle \partial_{t}v^{n},v\rangle \;dt\right)\\ & \;\;\leq \frac{1}{2}(\|v_{0}{\|}_{2}^{2}-\|v(T){\|}_{2}^{2})=\int_{0}^{T}\int_{\Omega }\text{S}:\text{D}v\;\;dx\;dt, \end{array}$$

where the last identity follows from (3.17) with setting w:=v. Consequently, using this estimate, the monotonicity and the growth assumption (1.6), the strong convergence result (3.14), the weak convergence (3.10) and the Lebesgue dominated convergence theorem, we deduce that for all $\bar{D}\in L^{p}(Q;R^{3\times 3}),$ there holds

3.21 $$\begin{array}{rl} 0 & \;\leq \underset{n\to \infty }{lim sup}\int_{0}^{T}\int_{\Omega }({\text{S}}^{n}-{\text{S}}^{*}(\vartheta^{n},\bar{\text{D}})):(\text{D}v^{n}-\bar{\text{D}})\;\;dx\;dt\\ & \;\leq \int_{0}^{T}\int_{\Omega }(\text{S}-{\text{S}}^{*}(\vartheta ,\bar{\text{D}})):(\text{D}v-\bar{\text{D}})\;\;dx\;dt. \end{array}$$

The classical Minty method then leads to (3.18). Moreover, setting $\bar{D}:=Dv$ in (3.21), we have

3.22 $$\underset{n\to \infty }{lim sup}\int_{0}^{T}\int_{\Omega }|({\text{S}}^{n}-{\text{S}}^{*}(\vartheta^{n},\text{D}v)):(\text{D}v^{n}-\text{D}v)|\;\;dx\;dt=0.$$

Hence,

3.23 $$({\text{S}}^{n}-{\text{S}}^{*}(\vartheta^{n},\text{D}v)):(\text{D}v^{n}-\text{D}v)\to 0\;\text{strongly in}\;L^{1}(Q).$$

Since

$${\text{S}}^{*}(\vartheta^{n},\text{D}v):(\text{D}v^{n}-\text{D}v)⇀0\;\text{weakly in}\;L^{1}(Q),$$

which follows from (3.10), (3.14) and (1.6*b*), we see that (3.23) directly implies (3.20).

### (b) Limit in entropy equation as n→+∞

In this section, we show the validity of (2.11). To see this, we first set $\psi :=\varphi /\vartheta^{n}$ in (3.4) with arbitrary $\varphi \in C_{0}^{\infty }((-\infty ,T)\times \Omega )$ to derive the following identity for approximated entropy $\eta^{n}:=\ln\vartheta^{n}$:

3.24 $$\begin{array}{rl} & \;-\int_{0}^{T}\int_{\Omega }\eta^{n}\partial_{t}\varphi +\eta^{n}\;v^{n}\cdot \nabla \varphi -\kappa (\vartheta^{n})\nabla \eta^{n}\cdot \nabla \varphi \;\;dx\;dt\\ & \;\;=\int_{0}^{T}\int_{\Omega }\frac{{\text{S}}^{n}:\text{D}v^{n}}{\vartheta^{n}}\;\varphi +\kappa (\vartheta^{n})\frac{|\nabla \vartheta^{n}{|}^{2}}{{\left(\vartheta^{n}\right)}^{2}}\;\varphi \;\;dx\;dt+\int_{\Omega }\eta_{0}^{n}\;\varphi (0)\;\;dx, \end{array}$$

where we set $\eta_{0}^{n}:=\ln\vartheta_{0}^{n}$. Next, we want to let n→∞. The identification of the limit in the terms on the left-hand side is rather standard and follows from the convergence results (3.10), (3.12), (3.14) and (3.15). Similarly, to pass to the limit in the first term on the right-hand side of (3.24) is straightforward thanks to (3.14), (3.20) and the Egorov and Dunford-Pettis theorems. Also the limit passage in the last term is obvious. The most problematic term is however the second term on the right-hand side since $\kappa (\vartheta^{n})|\nabla \vartheta^{n}{|}^{2}/{\left(\vartheta^{n}\right)}^{2}$ is uniformly bounded only in $L^{1}((0,T)\times \Omega ),$ and so we cannot even *a priori* extract an $L^{1}$ weakly convergent subsequence. We overcome this in two steps. First, the point-wise convergence of $\nabla \vartheta^{n}$ is shown, and then the strong convergence of $\kappa (\vartheta^{n})|\nabla \eta^{n}{|}^{2}$ in $L^{1}((0,T)\times \Omega )$ is deduced.

#### (i) Almost everywhere convergence of $\nabla \vartheta^{n}$

We start this part with definition of auxiliary cut-off functions. For arbitrary k>0, we define

3.25 $$T_{k}(z):=\mathrm{sign}(z)\min\{|z|,k\}.$$

Its primitive function attaining zero at zero is denoted $G_{k}$, i.e. $G_{k}^{\prime }=T_{k}$, $G_{k}(0)=0$. Note that $|G_{k}(s)|\leq k|s|$ for all s∈R. Next, we also introduce a mollification of $T_{k}$. For arbitrary δ∈(0,k) (typically δ≪1), we denote by $T_{k,\delta }\in C^{2}(R)$ a mollification of $T_{k}$, which is given by a convolution with a symmetric, positive kernel of radius δ. Such a mollification then has the following properties:

$$\begin{array}{rl} & T_{k,\delta }(z)=T_{k}(z)\;\text{ if }|z|\leq k-\delta \;\text{or}\;|z|\geq k+\delta ,\;|T_{k,\delta }^{{\;}^{\prime \prime }}|\leq C\delta^{-1}\\ & 0\leq T_{k,\delta }^{\prime }\leq 1,\;T_{k,\delta }^{{\;}^{\prime \prime }}\leq 0,\;T_{k,\delta }\leq T_{k}\;\text{on }(0,+\infty ). \end{array}$$

We fix m,n,k∈N, $k>2\|\hat{\vartheta }{\|}_{L^{\infty }(\partial \Omega )}$ and ε,δ>0, ε<k and define $w_{\delta }^{m,n}=T_{k+\varepsilon ,\delta }(\vartheta^{n})-T_{k,\delta }(\vartheta^{m})$. We set $\psi :=T_{k+\varepsilon ,\delta }^{\prime }(\vartheta^{n})T_{\varepsilon }(w_{\delta }^{m,n})$ in (3.4) for $\vartheta^{n}$ and $\psi :=T_{k,\delta }^{\prime }(\vartheta^{m})T_{\varepsilon }(w_{\delta }^{m,n})$ in (3.4) for $\vartheta^{m}$. Note that it is allowed since $T_{k+\varepsilon ,\delta }^{\prime }(\vartheta^{n})T_{\varepsilon }(w_{\delta }^{m,n})$ and also $T_{k,\delta }^{\prime }(\vartheta^{m})T_{\varepsilon }(w_{\delta }^{m,n})$ belong to $L^{2}((0,T);W_{0}^{1,2}(\Omega ))$. Then, we subtract the so obtained equations to obtain

3.26  ∫0T∫Ωκ(ϑn)∇(wδm,n)⋅∇Tε(wδm,n)  dx dt =∫0T∫Ω(κ(ϑn)−κ(ϑm))∇Tk,δ(ϑm)⋅∇Tε(wδm,n)  dx dt−∫0T⟨∂twδm,n,Tε(wδm,n)⟩ dt =+∫0T∫ΩGm,nTε(wδm,n)+[Tk+ε,δ(ϑn)vn−Tk,δ(ϑm)vm]⋅∇Tε(wδm,n)  dx dt,

where we denoted

$$\begin{array}{rl} G^{m,n} & \;:=[T_{k+\varepsilon ,\delta }^{\prime }(\vartheta^{n})\;{\text{S}}^{n}:\text{D}v^{n}-T_{k,\delta }^{\prime }(\vartheta^{m})\;{\text{S}}^{m}:\text{D}v^{m}]\\ & \;\;-[\kappa (\vartheta^{n})|\nabla \vartheta^{n}{|}^{2}T_{k+\varepsilon ,\delta }^{{\;}^{\prime \prime }}(\vartheta^{n})-\kappa (\vartheta^{m})|\nabla \vartheta^{m}{|}^{2}T_{k,\delta }^{{\;}^{\prime \prime }}(\vartheta^{m})]. \end{array}$$

Our first goal is to let $\delta \to 0_{+}$ in (3.26). Note that such convergence procedure is very standard in all terms except the term $G^{m,n}$ involving the second derivative of $T_{\cdot ,\delta }$. To obtain a proper δ-independent bound, we consider $M\geq \|\hat{\vartheta }{\|}_{L^{\infty }(\partial \Omega )}$ and δ∈(0,M/2). Since $\vartheta^{n}=\hat{\vartheta }$ on ∂Ω, we can deduce that $\psi :=1-T_{M,\delta }^{\prime }(\vartheta^{n})\in L^{2}(0,T;W_{0}^{1,2}(\Omega )),$ and therefore, it can be used in (3.4). Such a choice then leads to

3.27 $$\begin{array}{rl} & \;\int_{0}^{T}\int_{\Omega }\kappa (\vartheta^{n})|T_{M,\delta }^{{\;}^{\prime \prime }}(\vartheta^{n})\|\nabla \vartheta^{n}{|}^{2}\;\;dx\;dt=\int_{\Omega }(\vartheta_{0}^{n}-T_{M,\delta }(\vartheta_{0}^{n}))\;\;dx\\ & \;\;-\int_{\Omega }(\vartheta^{n}-T_{M,\delta }(\vartheta^{n}))(T)\;\;dx+\int_{0}^{T}\int_{\Omega }(1-T_{M,\delta }^{\prime }(\vartheta^{n})){\text{S}}^{n}:\text{D}v^{n}\;\;dx\;dt\\ & \;\;\leq \int_{\left\{\vartheta_{0}^{n}>M/2\right\}}\vartheta_{0}^{n}\;dx+\int_{\left\{|\vartheta^{n}|>M/2\right\}}{\text{S}}^{n}:\text{D}v^{n}\;dx\;dt\leq C, \end{array}$$

where we exploited that $\mathrm{div}v^{n}=0$, used the properties of $T_{M,\delta }$ (in particular the concavity) and (3.6) and (3.2). In a very similar manner, we can estimate the term with time derivative in (3.26). Since $\vartheta^{n}$ and $\vartheta^{m}$ belong for fix n,m to $C([0,T];L^{2}(\Omega ))$, we have (using the non-negativity of $G_{\varepsilon }$ as well as the estimate $G_{\varepsilon }(s)\leq s\varepsilon$)

3.28 $$\begin{array}{rl} & \;-\int_{0}^{T}\langle \partial_{t}w_{\delta }^{m,n},T_{\varepsilon }(w_{\delta }^{m,n})\rangle \;dt=-\int_{0}^{T}\int_{\Omega }\partial_{t}G_{\varepsilon }(w_{\delta }^{m,n})\;\;dx\;dt\leq \int_{\Omega }G_{\varepsilon }(w_{\delta }^{m,n}(0))\;\;dx\leq C\varepsilon . \end{array}$$

Hence, we can apply estimates (3.27) and (3.28) and then let $\delta \to 0_{+}$ in the remaining terms of (3.26) to deduce (recall $k\geq 2\|\hat{\vartheta }{\|}_{L^{\infty }(\partial \Omega )}$)

3.29 κ_∫0T∫Ω|∇Tε(wm,n)|2  dx dt≤∫0T∫Ω(κ(ϑn)−κ(ϑm))∇Tk(ϑm)⋅∇Tε(wm,n)  dx dt =+∫0T∫Ω[Tk+ε(ϑn)vn−Tk(ϑm)vm]⋅∇Tε(wm,n)  dx dt+Cε,

where $w^{m,n}:=T_{k+\varepsilon }(\vartheta^{n})-T_{k}(\vartheta^{m})$. The next goal is to let n,m→∞, and finally, $\varepsilon \to 0_{+}$. We start with the second term on the right-hand side. Defining $w_{\varepsilon }:=T_{k+\varepsilon }(\vartheta )-T_{k}(\vartheta )$ and using (3.12)–(3.15), we deduce

3.30 $$\begin{array}{rl} \lim_{\varepsilon \to 0_{+}} & \;\underset{n\to \infty }{lim sup}\underset{m\to \infty }{lim sup}\int_{0}^{T}\int_{\Omega }[T_{k+\varepsilon }(\vartheta^{n})v^{n}-T_{k}(\vartheta^{m})v^{m}]\cdot \nabla T_{\varepsilon }(w^{m,n})\;\;dx\;dt\\ & \;=\lim_{\varepsilon \to 0_{+}}\int_{0}^{T}\int_{\Omega }[T_{k+\varepsilon }(\vartheta )v-T_{k}(\vartheta )v]\cdot \nabla T_{\varepsilon }(w_{\varepsilon })\;\;dx\;dt=0. \end{array}$$

The remaining term in (3.29) is estimated with the help of (3.8) and the Hölder inequality as follows (note that $\left\{|w^{m,n}|<\epsilon \}\cap \{\vartheta^{m}\leq k\}=\{|\vartheta^{n}-\vartheta^{m}|<\epsilon \}\cap \{\vartheta^{m}\leq k\right\}$):

3.31 $$\begin{array}{rl} & \;\int_{0}^{T}\int_{\Omega }(\kappa (\vartheta^{n})-\kappa (\vartheta^{m}))\nabla T_{k}(\vartheta^{m})\cdot \nabla T_{\varepsilon }(w^{m,n})\;\;dx\;dt\\ & \;\;\leq 2\int_{0}^{T}\int_{\Omega }|\kappa (\vartheta^{n})-\kappa (\vartheta^{m})|(|\nabla \vartheta^{n}{|}^{2}+|\nabla \vartheta^{m}{|}^{2})\chi_{\left\{|w^{m,n}|<\varepsilon \}\cap \{\vartheta^{m}\leq k\right\}}\;\;dx\;dt\\ & \;\;\leq C(k)\||\kappa (\vartheta^{n})-\kappa (\vartheta^{m})|\chi_{\left\{|w^{m,n}|<\varepsilon \}\cap \{\vartheta^{m}\leq k\right\}}{\|}_{L^{\infty }(Q)}\\ & \;\;\leq C(k)\||\kappa (\vartheta^{n})-\kappa (\vartheta^{m})|\chi_{\left\{|\vartheta^{n}-\vartheta^{m}|<\varepsilon \}\cap \{\vartheta^{m}\leq k\right\}}{\|}_{L^{\infty }(Q)}\\ & \;\;\leq C(k)\underset{l,s\in [\mu ,2k];\;|s-l|\leq \varepsilon }{\sup}|\kappa (l)-\kappa (s)|\to 0 \end{array}$$

as $\varepsilon \to 0_{+}$ thanks to the uniform continuity of κ on [μ,2k].

Hence, using (3.30)–(3.31) in (3.29), we have

$$\lim_{\varepsilon \to 0_{+}}\underset{n\to \infty }{lim sup}\underset{m\to \infty }{lim sup}\int_{0}^{T}\int_{\Omega }|\nabla T_{\varepsilon }(w^{m,n}){|}^{2}\;\;dx\;dt=0,$$

which, thanks to the weak lower semicontinuity of the norm in $L^{2}(Q)$, gives

3.32 $$\lim_{\varepsilon \to 0_{+}}\underset{n\to \infty }{lim sup}\int_{0}^{T}\int_{\Omega }|\nabla T_{\varepsilon }(T_{k+\varepsilon }(\vartheta^{n})-T_{k}(\vartheta )){|}^{2}\;\;dx\;dt=0.$$

Finally, we use the Hölder inequality, the uniform bound (3.8) and the Tschebyschev inequality to obtain

$$\begin{array}{rl} & \;\int_{0}^{T}\int_{\Omega }|\nabla \vartheta^{n}-\nabla \vartheta |\;\;dx\;dt\leq \int_{0}^{T}\int_{\Omega }|\nabla T_{\varepsilon }(\vartheta^{n}-\vartheta )|\;\;dx\;dt\\ & \;\;+\int_{0}^{T}\int_{\Omega }|\nabla \vartheta^{n}-\nabla \vartheta |\chi_{\left\{|\vartheta^{n}-\vartheta |>\varepsilon \right\}}\;\;dx\;dt\\ & \;\;\leq \int_{0}^{T}\int_{\Omega }|\nabla T_{\varepsilon }(T_{k+\varepsilon }(\vartheta^{n})-T_{k}(\vartheta ))|\;\;dx\;dt\\ & \;\;+\int_{0}^{T}\int_{\Omega }|\nabla \vartheta^{n}-\nabla \vartheta |\chi_{\left\{|\vartheta^{n}-\vartheta |>\varepsilon \}\cup \{|\vartheta^{n}|+|\vartheta |>k/2\right\}}\;\;dx\;dt\\ & \;\;\leq C{\left(\int_{0}^{T}\int_{\Omega }|\nabla T_{\varepsilon }(T_{k+\varepsilon }(\vartheta^{n})-T_{k}(\vartheta )){|}^{2}\;\;dx\;dt\right)}^{\frac{1}{2}}\\ & \;\;+C\|\nabla \vartheta^{n}-\nabla \vartheta {\|}_{L^{9/8}(Q)}\left(|\{|\vartheta^{n}-\vartheta |>\varepsilon \}{|}^{\frac{1}{9}}+|\{|\vartheta^{n}|+|\vartheta |>k/2\}{|}^{\frac{1}{9}}\right)\\ & \;\;\leq C{\left(\int_{0}^{T}\int_{\Omega }|\nabla T_{\varepsilon }(T_{k+\varepsilon }(\vartheta^{n})-T_{k}(\vartheta )){|}^{2}\;\;dx\;dt\right)}^{\frac{1}{2}}+\frac{C\|\vartheta^{n}-\vartheta {\|}_{L^{1}(Q)}^{\frac{1}{9}}}{\varepsilon^{\frac{1}{9}}}+\frac{C}{k^{\frac{1}{9}}}. \end{array}$$

Therefore, the convergence result (3.14) leads to

$$\begin{array}{rl} & \;\lim_{n\to \infty }\int_{0}^{T}\int_{\Omega }|\nabla \vartheta^{n}-\nabla \vartheta |\;\;dx\;dt\leq C{\left(\underset{n\to \infty }{lim sup}\int_{0}^{T}\int_{\Omega }|\nabla T_{\varepsilon }(T_{k+\varepsilon }(\vartheta^{n})-T_{k}(\vartheta )){|}^{2}\;\;dx\;dt\right)}^{1/2}+\frac{C}{k^{1/9}}. \end{array}$$

As the left-hand side is independent of ε and k, we may let first $\varepsilon \to 0_{+}$ and use (3.32) to eliminate the first term on the right-hand side and then let k→∞ to handle the second term on the right-hand side and thus to observe that

3.33 $$\nabla \vartheta^{n}\to \nabla \vartheta \;\text{strongly in}\;L^{1}(Q),$$

and consequently (for a subsequence)

3.34 $$\nabla \vartheta^{n}\to \nabla \vartheta \;\text{a.e. in}\;Q.$$

#### (ii) Strong convergence of $\kappa (\vartheta^{n})|\nabla \vartheta^{n}{|}^{2}/{\left(\vartheta^{n}\right)}^{2}$n in $L^{1}$-norm

We start the proof of the claim by showing a strong convergence of $\nabla T_{k}(\vartheta^{n}-\hat{\vartheta })$ in $L^{2}(Q)$ for arbitrary k. To prove such a result, we want to set $\psi :=T_{k}(\vartheta^{n}-\hat{\vartheta })$ in (3.4), let n→∞ and compare the limit with (2.10) tested by $\varphi :=T_{k}(\vartheta -\hat{\vartheta })$. However, such test functions are not allowed in general, and therefore, we must proceed more carefully. We fix an arbitrary $T^{*}\in (0,T)$, which is the Lebesgue point of ϑ(t) as a function in $L^{1}(0,T;L^{1}(\Omega ))$. By using the fact that $\mathrm{div}v^{n}=0$, we see that

3.35 $$\int_{\Omega }\nabla \vartheta^{n}\cdot v^{n}\;T_{k}(\vartheta^{n}-\hat{\vartheta })\;\;dx=\int_{\Omega }\nabla \hat{\vartheta }\cdot v^{n}\;T_{k}(\vartheta^{n}-\hat{\vartheta })\;\;dx.$$

Then, we set $\psi :=T_{k}(\vartheta^{n}-\hat{\vartheta })\chi_{\left[0,\tau \right]}$ with $\tau \in (T^{*},T)$ arbitrary in (3.4). We deduce (since $\vartheta^{n}\in C([0,T];L^{2}(\Omega ))$ and $\hat{\vartheta }$ is independent of time) by using (3.35) that

3.36 $$\begin{array}{rl} & \;\int_{0}^{\tau }\int_{\Omega }\kappa (\vartheta^{n})\nabla (\vartheta^{n}-\hat{\vartheta })\cdot \nabla [T_{k}(\vartheta^{n}-\hat{\vartheta })]\;\;dx\;dt=-\int_{\Omega }G_{k}(\vartheta^{n}(\tau )-\hat{\vartheta })-G_{k}(\vartheta_{0}^{n}-\hat{\vartheta })\;\;dx\\ & \;\;+\int_{0}^{\tau }\int_{\Omega }-\nabla \hat{\vartheta }\cdot v^{n}\;T_{k}(\vartheta^{n}-\hat{\vartheta })-\kappa (\vartheta^{n})\nabla \hat{\vartheta }\cdot \nabla [T_{k}(\vartheta^{n}-\hat{\vartheta })]+T_{k}(\vartheta^{n}-\hat{\vartheta })\;{\text{S}}^{n}:\text{D}v^{n}\;\;dx\;dt. \end{array}$$

Then, it follows from (3.12), (3.14), (3.2), (3.20) and (3.36) that for $\delta \in (0,T-T^{*})$

3.37 $$\begin{array}{rl} & \;\underset{n\to \infty }{lim sup}\int_{0}^{T^{*}}\int_{\Omega }\kappa (\vartheta^{n})\nabla \vartheta^{n}\cdot \nabla [T_{k}(\vartheta^{n}-\hat{\vartheta })]\;\;dx\;dt\\ & \;\;=\underset{n\to \infty }{lim sup}\int_{0}^{T^{*}}\int_{\Omega }\kappa (\vartheta^{n})\nabla [\vartheta^{n}-\hat{\vartheta }]\cdot \nabla [T_{k}(\vartheta^{n}-\hat{\vartheta })]\\ & \;\;+\kappa (\vartheta^{n})\nabla \hat{\vartheta }\cdot \nabla [T_{k}(\vartheta^{n}-\hat{\vartheta })]\;\;dx\;dt\\ & \;\;\leq \underset{n\to \infty }{lim sup}{\text{⨏}}_{T^{*}}^{T^{*}+\delta }\int_{0}^{\tau }\int_{\Omega }\kappa (\vartheta^{n})\nabla [\vartheta^{n}-\hat{\vartheta }]\cdot \nabla [T_{k}(\vartheta^{n}-\hat{\vartheta })]\;\;dx\;dt\;d\tau \\ & \;\;\;+\int_{0}^{T^{*}}\int_{\Omega }\kappa (\vartheta )\nabla \hat{\vartheta }\cdot \nabla [T_{k}(\vartheta -\hat{\vartheta })]\;\;dx\;dt\\ & \;\;\overset{\left(3.36\right)}{\to }-\int_{\Omega }G_{k}(\vartheta (T^{*})-\hat{\vartheta })-G_{k}(\vartheta_{0}-\hat{\vartheta })\;\;dx\\ & \;\;\;+\int_{0}^{T^{*}}\int_{\Omega }(\text{S}:\text{D}v-\nabla \hat{\vartheta }\cdot v)T_{k}(\vartheta -\hat{\vartheta })\;\;dx\;dt \end{array}$$

as $\delta \to 0_{+}$. Now, the aim is to identify the final expression in (3.37). We want to set $\psi :=T_{k}(\vartheta -\hat{\vartheta })$ in (3.19). Unfortunately, it is not possible due to low regularity of ϑ. Therefore, we must proceed differently. In what follows, we always consider M,k,ε,δ>0 such that $M>k+1+\|\hat{\vartheta }{\|}_{\infty }$ and δ∈(0,1). Due to the smoothness of $\vartheta^{n}$, we can set $\psi (\tau ,x):=T_{M,\delta }^{\prime }(\vartheta^{n}(\tau ,x))\chi_{\left(t,t+\varepsilon \right)}(\tau )\varphi (x)$, τ∈(0,T), x∈Ω in (3.4) to deduce that for all $\varphi \in W_{0}^{1,2}(\Omega )\cap L^{\infty }(\Omega )$ and all $t\in (0,T^{*})$, $\varepsilon \in (0,T-T^{*})$ we have

$$\begin{array}{rl} & \;\int_{\Omega }\partial_{t}{\text{⨏}}_{t}^{t+\varepsilon }T_{M,\delta }(\vartheta^{n})\;d\tau \varphi \;\;dx+\int_{\Omega }{\text{⨏}}_{t}^{t+\varepsilon }\nabla [T_{M,\delta }(\vartheta^{n})]\cdot v^{n}\;d\tau \varphi \;\;dx\\ & \;\;+\int_{\Omega }{\text{⨏}}_{t}^{t+\varepsilon }\kappa (\vartheta^{n})\nabla T_{M,\delta }(\vartheta^{n})\;d\tau \cdot \nabla \varphi \;\;dx=\int_{\Omega }{\text{⨏}}_{t}^{t+\varepsilon }T_{M,\delta }^{\prime }(\vartheta^{n})\;{\text{S}}^{n}:\text{D}v^{n}\;d\tau \varphi \;\;dx\\ & \;\;+\int_{\Omega }{\text{⨏}}_{t}^{t+\varepsilon }\kappa (\vartheta^{n})T_{M,\delta }^{{\;}^{\prime \prime }}(\vartheta^{n})|\nabla \vartheta^{n}{|}^{2}\;d\tau \varphi \;\;dx. \end{array}$$

Then, we fix a measurable set $J\subset (0,T^{*})$ and integrate over t∈J. By using the convergence results (3.12)–(3.14) and (3.20) and the density of step functions in $L^{2}(0,T^{*};W_{0}^{1,2}(\Omega ))\cap L^{\infty }(0,T;L^{\infty }(\Omega ))$, we obtain that for all $\varphi \in L^{2}(0,T;W_{0}^{1,2}(\Omega ))\cap L^{\infty }(Q)$,

3.38 $$\begin{array}{rl} & \;\int_{0}^{T^{*}}\int_{\Omega }\partial_{t}\vartheta_{\varepsilon }^{M,\delta }\varphi \;\;dx\;dt+\int_{0}^{T^{*}}\int_{\Omega }{\text{⨏}}_{t}^{t+\varepsilon }\nabla [T_{M,\delta }(\vartheta )]\cdot v\;d\tau \varphi \;\;dx\;dt\\ & \;\;\;+\int_{0}^{T^{*}}\int_{\Omega }{\text{⨏}}_{t}^{t+\varepsilon }\kappa (\vartheta )\nabla T_{M,\delta }(\vartheta )\;d\tau \cdot \nabla \varphi \;\;dx\;dt\\ & \;\;=\int_{0}^{T^{*}}\int_{\Omega }{\text{⨏}}_{t}^{t+\varepsilon }T_{M,\delta }^{\prime }(\vartheta )\;\text{S}:\text{D}v\;d\tau \varphi \;\;dx\;dt\\ & \;\;\;+\underset{n\to \infty }{lim sup}\int_{0}^{T^{*}}\int_{\Omega }{\text{⨏}}_{t}^{t+\varepsilon }\kappa (\vartheta^{n})T_{M,\delta }^{{\;}^{\prime \prime }}(\vartheta^{n})|\nabla \vartheta^{n}{|}^{2}\;d\tau \varphi \;\;dx\;dt, \end{array}$$

where we denoted $\vartheta_{\varepsilon }^{M,\delta }(t,x):={\text{⨏}}_{t}^{t+\varepsilon }T_{M,\delta }(\vartheta (\tau ,x))\;d\tau$. Note that for every ε>0, the function $\vartheta_{\varepsilon }^{M,\delta }$ belongs to $W^{1,\infty }(0,T;L^{\infty }(\Omega ))$ and so the integral with time derivative is well defined. From (3.27), (3.2), (3.14) and (3.20), we obtain

3.39 $$\begin{array}{rl} & \;\underset{n\to \infty }{lim sup}\int_{0}^{T}\int_{\Omega }\kappa (\vartheta^{n})|T_{M,\delta }^{{\;}^{\prime \prime }}(\vartheta^{n})\|\nabla \vartheta^{n}{|}^{2}\;\;dx\;dt\\ & \;\;\leq \int_{\Omega }(\vartheta_{0}-T_{M,\delta }(\vartheta_{0}))\;\;dx+\int_{0}^{T}\int_{\Omega }(1-T_{M,\delta }^{\prime }(\vartheta ))\text{S}:\text{D}v\;\;dx\;dt\\ & \;\;\leq \int_{\left\{\vartheta_{0}>M/2\right\}}\vartheta_{0}\;dx+\int_{\left\{|\vartheta |>M/2\right\}}\text{S}:\text{D}v\;dx\;dt. \end{array}$$

By setting $\varphi =T_{k}(\vartheta_{\varepsilon }^{M,\delta }-\hat{\vartheta })$ in (3.38) and using (3.39), we obtain

$$\begin{array}{rl} & \;\int_{0}^{T^{*}}\int_{\Omega }{\text{⨏}}_{t}^{t+\varepsilon }\kappa (\vartheta )\nabla T_{M,\delta }(\vartheta )\;d\tau \cdot \nabla T_{k}(\vartheta_{\varepsilon }^{M,\delta }-\hat{\vartheta })\;\;dx\;dt\\ & \;\;\geq -\int_{\Omega }G_{k}(\vartheta_{\varepsilon }^{M,\delta }(T^{*})-\hat{\vartheta })-G_{k}(\vartheta_{\varepsilon }^{M,\delta }(0)-\hat{\vartheta })\;\;dx\\ & \;\;\;+\int_{0}^{T^{*}}\int_{\Omega }({\text{⨏}}_{t}^{t+\varepsilon }T_{M,\delta }^{\prime }(\vartheta )\;\text{S}:\text{D}v-\nabla [T_{M,\delta }(\vartheta )]\cdot v\;d\tau )T_{k}(\vartheta_{\varepsilon }^{M,\delta }-\hat{\vartheta })\;\;dx\;dt\\ & \;\;\;-k\left(\int_{\left\{\vartheta_{0}>M/2\right\}}\vartheta_{0}\;dx+\int_{\left\{\vartheta >M/2\right\}}\text{S}:\text{D}v\;dx\;dt\right). \end{array}$$

Since $T^{*}$ is the Lebesgue point of ϑ and since the initial condition is attained (see e.g. [1,3]), we can let $\varepsilon \to 0_{+}$ and obtain

3.40 $$\begin{array}{rl} & \;\int_{0}^{T^{*}}\int_{\Omega }\kappa (\vartheta )\nabla T_{M,\delta }(\vartheta )\cdot \nabla T_{k}(T_{M,\delta }(\vartheta )-\hat{\vartheta })\;\;dx\;dt\\ & \;\;\geq -\int_{\Omega }G_{k}(T_{M,\delta }(\vartheta )(T^{*})-\hat{\vartheta })-G_{k}(T_{M,\delta }(\vartheta_{0})-\hat{\vartheta })\;\;dx\\ & \;\;\;+\int_{0}^{T^{*}}\int_{\Omega }(T_{M,\delta }^{\prime }(\vartheta )\;\text{S}:\text{D}v-\nabla [T_{M,\delta }(\vartheta )]\cdot v)T_{k}(T_{M,\delta }(\vartheta )-\hat{\vartheta })\;\;dx\;dt\\ & \;\;\;-k\left(\int_{\left\{\vartheta_{0}>M/2\right\}}\;\vartheta_{0}\;dx+\int_{\left\{\vartheta >M/2\right\}}\;\text{S}:\text{D}v\;dx\;dt\right). \end{array}$$

For $M>k+1+\|\hat{\vartheta }{\|}_{\infty }$, we have $T_{k}(T_{M,\delta }(\vartheta )-\hat{\vartheta })=T_{k}(\vartheta -\hat{\vartheta })$; thus, one can let M→∞ in (3.40). By using integration by parts and the fact that div⁡v=0 similarly as in (3.35), one obtains

$$\begin{array}{rl} & \;\int_{0}^{T^{*}}\int_{\Omega }\kappa (\vartheta )\nabla \vartheta \cdot \nabla T_{k}(\vartheta -\hat{\vartheta })\;\;dx\;dt\\ & \;\;\geq -\int_{\Omega }G_{k}(\vartheta (T^{*})-\hat{\vartheta })-G_{k}(\vartheta_{0}-\hat{\vartheta })\;\;dx+\int_{0}^{T^{*}}\int_{\Omega }(\text{S}:\text{D}v-\nabla \hat{\vartheta }\cdot v)T_{k}(\vartheta -\hat{\vartheta })\;\;dx\;dt. \end{array}$$

Comparing the result with (3.37), we see that

3.41 $$\underset{n\to +\infty }{lim sup}\int_{0}^{T^{*}}\int_{\Omega }\kappa (\vartheta^{n})\nabla \vartheta^{n}\cdot \nabla T_{k}(\vartheta^{n}-\hat{\vartheta })\;\;dx\;dt\leq \int_{0}^{T^{*}}\int_{\Omega }\kappa (\vartheta )\nabla \vartheta \cdot \nabla T_{k}(\vartheta -\hat{\vartheta })\;\;dx\;dt,$$

which is the cornerstone for the strong convergence. Indeed, it follows from (3.13), (3.14) and from (3.41) that (using also the Vitali convergence theorem and (1.5))

3.42 $$\underset{n\to +\infty }{lim sup}\int_{0}^{T^{*}}\int_{\Omega }\kappa (\vartheta^{n})|\nabla T_{k}(\vartheta^{n}-\hat{\vartheta }){|}^{2}\;\;dx\;dt\leq \int_{0}^{T^{*}}\int_{\Omega }\kappa (\vartheta )|\nabla T_{k}(\vartheta -\hat{\vartheta }){|}^{2}\;\;dx\;dt.$$

Since

3.43 $$\sqrt{\kappa (\vartheta^{n})}\nabla T_{k}(\vartheta^{n}-\hat{\vartheta })⇀\sqrt{\kappa (\vartheta )}\nabla T_{k}(\vartheta -\hat{\vartheta })\;\text{weakly in}\;L^{2}(Q),$$

the inequality (3.42), weak lower semicontinuity of $L^{2}$ norm and uniform convexity of $L^{2}$ imply

3.44 $$\sqrt{\kappa (\vartheta^{n})}\nabla T_{k}(\vartheta^{n}-\hat{\vartheta })\to \sqrt{\kappa (\vartheta )}\nabla T_{k}(\vartheta -\hat{\vartheta })\;\text{strongly in}\;L^{2}((0,T^{*})\times \Omega ).$$

Finally, we want to show that

3.45 $$\sqrt{\kappa (\vartheta^{n})}\frac{\nabla \vartheta^{n}}{\vartheta^{n}}\to \sqrt{\kappa (\vartheta )}\frac{\nabla \vartheta }{\vartheta }\;\text{strongly in}\;L^{2}((0,T^{*})\times \Omega ).$$

We have

3.46 $$\sqrt{\kappa (\vartheta^{n})}\frac{\nabla \vartheta^{n}}{\vartheta^{n}}⇀\sqrt{\kappa (\vartheta )}\frac{\nabla \vartheta }{\vartheta }\;\text{weakly in}\;L^{2}((0,T^{*})\times \Omega )$$

because of the uniform boundedness in $L^{2}((0,T^{*})\times \Omega ).$ To show convergence of norms, we write

3.47 $$\begin{array}{rl} & \;\int_{0}^{T^{*}}\int_{\Omega }\kappa (\vartheta^{n})\frac{|\nabla \vartheta^{n}{|}^{2}}{{\left(\vartheta^{n}\right)}^{2}}\;\;dx\;dt\\ & \;\;=\int_{0}^{T^{*}}\int_{\Omega }\kappa (\vartheta^{n})\frac{\nabla \vartheta^{n}}{\vartheta^{n}}\cdot \frac{\nabla (\vartheta^{n}-\hat{\vartheta })}{\vartheta^{n}}\;\;dx\;dt+\int_{0}^{T^{*}}\int_{\Omega }\kappa (\vartheta^{n})\frac{\nabla \vartheta^{n}}{\vartheta^{n}}\cdot \frac{\nabla \hat{\vartheta }}{\vartheta^{n}}\;\;dx\;dt\\ & \;\;=\int_{0}^{T^{*}}\int_{\Omega }\kappa (\vartheta^{n})\frac{\nabla \vartheta^{n}}{\vartheta^{n}}\cdot \frac{\nabla T_{k}(\vartheta^{n}-\hat{\vartheta })}{\vartheta^{n}}\;\;dx\;dt+\int_{\left\{|\vartheta^{n}-\hat{\vartheta }|>k\right\}}\;\kappa (\vartheta^{n})\frac{\nabla \vartheta^{n}}{\vartheta^{n}}\cdot \frac{\nabla (\vartheta^{n}-\hat{\vartheta })}{\vartheta^{n}}\;dx\;dt\\ & \;\;\;+\int_{0}^{T^{*}}\int_{\Omega }\kappa (\vartheta^{n})\frac{\nabla \vartheta^{n}}{\vartheta^{n}}\cdot \frac{\nabla \hat{\vartheta }}{\vartheta^{n}}\;\;dx\;dt. \end{array}$$

Next, we let n→∞ on the right-hand side of (3.47). First, by the weak convergence (3.46), the strong convergence results (3.14) and (3.44), the Vitali theorem and the minimum principle $\vartheta^{n}\geq \mu ,$

$$\lim_{n\to +\infty }\int_{0}^{T^{*}}\int_{\Omega }\kappa (\vartheta^{n})\frac{\nabla \vartheta^{n}}{\vartheta^{n}}\cdot \frac{\nabla T_{k}(\vartheta^{n}-\hat{\vartheta })}{\vartheta^{n}}\;\;dx\;dt=\int_{0}^{T^{*}}\int_{\Omega }\kappa (\vartheta )\frac{\nabla \vartheta }{\vartheta }\cdot \frac{\nabla T_{k}(\vartheta -\hat{\vartheta })}{\vartheta }\;\;dx\;dt$$

and

3.48 $$\lim_{n\to +\infty }\int_{0}^{T^{*}}\int_{\Omega }\kappa (\vartheta^{n})\frac{\nabla \vartheta^{n}}{\vartheta^{n}}\cdot \frac{\nabla \hat{\vartheta }}{\vartheta^{n}}\;\;dx\;dt=\int_{0}^{T^{*}}\int_{\Omega }\kappa (\vartheta )\frac{\nabla \vartheta }{\vartheta }\cdot \frac{\nabla \hat{\vartheta }}{\vartheta }\;\;dx\;dt.$$

In addition, assuming that $k>\|\hat{\vartheta }{\|}_{\infty }$, we see that $\vartheta^{n}\geq k$ on the set $\left\{|\vartheta^{n}-\hat{\vartheta }|>k\right\}$. Consequently, with the help of the Young inequality, we deduce that for any λ∈(0,1)

3.49 $$\begin{array}{rl} & \left|\int_{\left\{|\vartheta^{n}-\hat{\vartheta }|>k\right\}}\kappa (\vartheta^{n})\frac{\nabla \vartheta^{n}}{\vartheta^{n}}\cdot \frac{\nabla (\vartheta^{n}-\hat{\vartheta })}{\vartheta^{n}}\;dx\;dt\right|\leq 2\bar{\kappa }\int_{0}^{T}\int_{\Omega }\frac{|\nabla \vartheta^{n}{|}^{2}+|\nabla \hat{\vartheta }{|}^{2}}{{\left(\vartheta^{n}\right)}^{1+\lambda }k^{1-\lambda }}\;\;dx\;dt\leq \frac{C(\lambda )}{k^{1-\lambda }}, \end{array}$$

where C is independent of n and k thanks to the uniform estimate in (3.8) and the minimum principle $\vartheta^{n}\geq \mu$. Equality (3.47) and inequality (3.49) remain valid also if we replace $\vartheta^{n}$ with ϑ. Consequently, it follows

3.50 $$\underset{n\to \infty }{lim sup}\int_{0}^{T^{*}}\int_{\Omega }\kappa (\vartheta^{n})\frac{|\nabla \vartheta^{n}{|}^{2}}{{\left(\vartheta^{n}\right)}^{2}}\;\;dx\;dt\leq \int_{0}^{T^{*}}\int_{\Omega }\kappa (\vartheta )\frac{|\nabla \vartheta {|}^{2}}{\vartheta^{2}}\;\;dx\;dt+\frac{C(\lambda )}{k^{1-\lambda }}.$$

Since $k>\|\hat{\vartheta }{\|}_{\infty }$ is arbitrary, (3.50) gives convergence of norms, which combined with (3.46) implies the strong convergence (3.45). Finally, let us recall that $T^{*}$ can be chosen arbitrarily close to T. In addition, we can *a priori* construct the solution on the time interval (0,2T). Consequently, $T^{*}$ can be chosen bigger than T, and so we obtain (3.45) with $T^{*}$ replaced by T.

#### (iii) Limit in the entropy equality

Let us analyze the limit of (3.24) as n→+∞ for each term. The strong convergence of $\vartheta^{n}$ and $\nabla \vartheta^{n}$, see (3.14) and (3.33), implies that $\eta^{n}\to \eta$ and $\nabla \eta^{n}\to \nabla \eta$ a.e. in Q. Thanks to the definition of $\eta^{n}$, and the *a priori* bound (3.8), we obtain a uniform bound for $\|\eta^{n}{\|}_{L^{r}(Q)}$ for any r∈(1,+∞) and for $\|\nabla \eta^{n}{\|}_{L^{2}(Q)}$. These facts, the Vitali convergence theorem and (3.2) allow us, up to subsequence, to easily pass to the limit on the left-hand side of (3.24).

To pass to the limit also in terms on the right-hand side, we use (3.10), (3.11), (3.14), (3.23) and (3.45) combined with the Lebesgue dominated convergence theorem and together with the minimum principle for $\vartheta^{n}$. Consequently, η satisfies entropy equation (2.11).

### (c) Continuity of ϑ in time

Finally, we focus on the attainment of initial conditions and continuity with respect to time variable. Concerning the velocity field, we can recall (3.10) and by standard parabolic interpolation, we observe $v\in C([0,T];L_{0,\mathrm{div}}^{2})$. The fact that $v(0)=v_{0}$ is then proven analogously as for Navier–Stokes equations, see e.g. [2,8].

Now, we focus on the temperature. For the attainment of the initial condition, one can follow [1–3]. Thus, we present here only the proof of continuity of ϑ with respect to time into the $L^{1}$ topology. The key idea is the following. We investigate a function (here $M>\max\{\|\hat{\vartheta }{\|}_{L^{\infty }(\Omega )},2,2\mu \}$)

$$g(\vartheta -\hat{\vartheta }):=\mathrm{sign}(\vartheta -\hat{\vartheta })\sqrt{G_{M}(\vartheta -\hat{\vartheta })}$$

and show first that for all $\psi \in L^{2}(\Omega ),$ the mapping $t\mapsto \int_{\Omega }g(\vartheta (t,x)-\hat{\vartheta }(t,x))\psi (x)\;\;dx$ is continuous on [0,T]. Second, we show that the mapping $t\mapsto \int_{\Omega }g^{2}(\vartheta (t,x)-\hat{\vartheta }(t,x))\;\;dx$ is continuous on [0,T]. As a direct consequence of these two properties, we obtain that

3.51 $$g(\vartheta -\hat{\vartheta })\in C([0,T];L^{2}(\Omega )).$$

Since

$$g^{\prime }(\vartheta -\hat{\vartheta })=\frac{|T_{M}(\vartheta -\hat{\vartheta })|}{2\sqrt{G_{M}(\vartheta -\hat{\vartheta })}}=\left\{\begin{array}{ll} \frac{1}{\sqrt{2}}, & \text{if }\vartheta \leq M+\hat{\vartheta }\\ \frac{M}{2\sqrt{M(\vartheta -\hat{\vartheta })-\frac{M^{2}}{2}}} & \text{if }\vartheta \geq M+\hat{\vartheta } \end{array}\right\}\geq \frac{\sqrt{\mu }}{\sqrt{2\vartheta }},$$

we have a trivial estimate

$$|\vartheta_{1}-\vartheta_{2}|\leq \sqrt{2}(\sqrt{\vartheta_{1}}+\sqrt{\vartheta_{2}})|g(\vartheta_{1}-\hat{\vartheta })-g(\vartheta_{2}-\hat{\vartheta })|.$$

Consequently, by using the Hölder inequality and (3.16), we observe

$$\begin{array}{rl} \|\vartheta (t_{1})-\vartheta (t_{2}){\|}_{L^{1}(\Omega )} & \;\leq \sqrt{2}\|\sqrt{\vartheta (t_{1})}+\sqrt{\vartheta (t_{2})}{\|}_{L^{2}(\Omega )}\|g(\vartheta (t_{1})-\hat{\vartheta })-g(\vartheta (t_{2})-\hat{\vartheta }){\|}_{L^{2}(\Omega )}\\ & \;\leq C\|g(\vartheta (t_{1})-\hat{\vartheta })-g(\vartheta (t_{2})-\hat{\vartheta }){\|}_{L^{2}(\Omega )}. \end{array}$$

Thus, we see that (3.51) implies $\vartheta \in C([0,T];L^{1}(\Omega ))$.

It remains to show (3.51). Firstly, we set $\psi :=g^{\prime }(\vartheta^{n}-\hat{\vartheta })\chi_{\left[0,\tau \right]}\varphi$ with $\varphi \in C_{0}^{1}(\Omega )$ arbitrary in (3.4). By using integration by parts, we have

$$\begin{array}{rl} & \;\int_{\Omega }(g(\vartheta^{n}(\tau )-\hat{\vartheta })-g(\vartheta^{n}(0)-\hat{\vartheta }))\varphi \;\;dx=-\int_{0}^{\tau }\int_{\Omega }g^{\prime }(\vartheta^{n}-\hat{\vartheta })\varphi v^{n}\nabla \hat{\vartheta }\;\;dx\;dt\\ & \;\;+\int_{0}^{\tau }\int_{\Omega }(v^{n}(\vartheta^{n}-\hat{\vartheta })-\kappa (\vartheta^{n})\nabla \vartheta^{n})\cdot (\varphi g^{\prime \prime }(\vartheta^{n}-\hat{\vartheta })\nabla (\vartheta^{n}-\hat{\vartheta })+g^{\prime }(\vartheta^{n}-\hat{\vartheta })\nabla \varphi )\;\;dx\;dt\\ & \;\;+\int_{0}^{\tau }\int_{\Omega }{\text{S}}^{n}:\text{D}v^{n}g^{\prime }(\vartheta^{n}-\hat{\vartheta })\varphi \;\;dx\;dt. \end{array}$$

Next, we let n→∞ in the above identity. By using the uniform estimates (3.6)–(3.8), the properties of g, the convergence results (3.10), (3.14), (3.34), (3.46) and the Vitali convergence theorem, we can easily identify the limits in the first two terms on the right-hand side. For the last term on the right-hand side, we also use (3.20). In addition, for almost all τ∈(0,T), we can also identify the limit on the left-hand side:

3.52 $$\begin{array}{rl} & \;\int_{\Omega }(g(\vartheta (\tau )-\hat{\vartheta })-g(\vartheta_{0}-\hat{\vartheta }))\varphi \;\;dx=-\int_{0}^{\tau }\int_{\Omega }g^{\prime }(\vartheta -\hat{\vartheta })\varphi v\nabla \hat{\vartheta }\;\;dx\;dt\\ & \;\;+\int_{0}^{\tau }\int_{\Omega }(v(\vartheta -\hat{\vartheta })-\kappa (\vartheta )\nabla \vartheta )\cdot (\varphi g^{\prime \prime }(\vartheta -\hat{\vartheta })\nabla (\vartheta -\hat{\vartheta })+g^{\prime }(\vartheta -\hat{\vartheta })\nabla \varphi )\;\;dx\;dt\\ & \;\;+\int_{0}^{\tau }\int_{\Omega }\text{S}:\text{D}vg^{\prime }(\vartheta -\hat{\vartheta })\varphi \;\;dx\;dt. \end{array}$$

Since the right-hand side is a continuous function of τ∈[0,T], we can redefine $g(\vartheta -\hat{\vartheta })$ on zero subset of [0,T] to obtain

3.53 $$\left(\int_{\Omega }g(\vartheta )\varphi \;\;dx\right)\in C([0,T])\;\text{for all}\;\varphi \in C_{0}^{1}(\Omega ).$$

Since $g\in L^{\infty }(0,T;L^{2}(\Omega ))$ and $C_{0}^{1}(\Omega )$ is dense in $L^{2}(\Omega )$, (3.53) implies that

3.54 $$\left(\int_{\Omega }g(\vartheta )\varphi \;\;dx\right)\in C([0,T])\;\text{for all}\;\varphi \in L^{2}(\Omega ).$$

Finally, we pass in (3.36) to the limit as n→+∞ similarly as in (3.37) using also (3.46)

3.55 $$\begin{array}{rl} & \;\int_{\Omega }G_{M}(\vartheta (\tau )-\hat{\vartheta })-G_{M}(\vartheta_{0}-\hat{\vartheta })\;\;dx=\int_{0}^{\tau }\int_{\Omega }-\kappa (\vartheta )\nabla \vartheta \cdot \nabla [T_{M}(\vartheta -\hat{\vartheta })]\;\;dx\;dt\\ & \;\;+\int_{0}^{\tau }\int_{\Omega }-\nabla \hat{\vartheta }\cdot v\;T_{M}(\vartheta -\hat{\vartheta })+T_{M}(\vartheta -\hat{\vartheta })\;\text{S}:\text{D}v\;\;dx\;dt=:\int_{0}^{\tau }\int_{\Omega }h\;\;dx\;dt, \end{array}$$

where $h\in L^{1}(Q)$. Hence, we see that (it can be continuously extended)

$$\int_{\Omega }G_{M}(\vartheta (\tau )-\hat{\vartheta })\;\;dx\in C([0,T]).$$

Since ${\left(g(\theta -\hat{\vartheta })\right)}^{2}=G_{M}(\vartheta -\hat{\vartheta })$, the aforementioned relation combined with (3.54) implies (3.51). The proof is^1^ complete.

## Data Availability

This article has no additional data.

## Footnotes

1 In fact in the aforementioned procedure, we somehow extended g and $G_{M}$ also to possible non-Lebesgue points. This can be done more carefully. Namely, one can consider ${\text{⨏}}_{t}^{t+\delta }g(\tau )\;d\tau$ and ${\text{⨏}}_{t}^{t+\delta }G_{M}(\vartheta (\tau )-\hat{\vartheta })$. These are surely continuous with respect to t∈[0,T]. Then thanks to (3.54) and (3.55), we see that

$${\text{⨏}}_{t}^{t+\delta }g(\tau )\;d\tau \to g\;\text{strongly in }C([0,T];L^{2}(\Omega )).$$
